# Staged Transmediastinal Drainage Followed by Robot-Assisted Thoracoscopic Resection for a Giant Anterior Mediastinal Cystic Teratoma: A Case Report

**DOI:** 10.5761/atcs.cr.26-00110

**Published:** 2026-09-10

**Authors:** Yuki Yamada, Tomohiro Haruki, Naoyuki Oka, Masaya Yotsukura, Yukihiro Yoshida, Shun-Ichi Watanabe

**Affiliations:** Department of Thoracic Surgery, National Cancer Center Hospital, Tokyo, Japan

**Keywords:** anterior mediastinal tumor, cystic teratoma, preoperative drainage, robot-assisted thoracoscopic surgery, minimally invasive surgery

## Abstract

Giant cystic anterior mediastinal tumors pose a significant challenge for minimally invasive surgery because of limited working space and the risk of cyst rupture. A 26-year-old woman presented with a large anterior mediastinal cystic tumor strongly suggestive of a benign lesion on imaging. To secure an adequate operative field and reduce the risk of intraoperative rupture, computed tomography–guided transmediastinal drainage was performed preoperatively. Tumor volume reduction improved surgical exposure and maneuverability and reduced the risk of rupture, enabling robot-assisted thoracoscopic surgery. Despite dense inflammatory adhesions between the tumor and surrounding tissues, precise dissection was achieved with robotic assistance, allowing complete tumor resection without rupture. This case suggests that a staged strategy combining preoperative drainage and robotic surgery may represent a safe and effective minimally invasive approach for selected patients with giant benign mediastinal cystic tumors.

## Introduction

Giant cystic mediastinal tumors pose a technical challenge for minimally invasive surgery because of limited working space and the risk of spillage of cystic contents due to tumor rupture; therefore, conventional open approaches such as median sternotomy have traditionally been selected. In recent years, several reports have demonstrated that aspiration of cystic contents can reduce tumor volume, improve surgical exposure and maneuverability, and facilitate thoracoscopic resection. Furthermore, robot-assisted thoracoscopic surgery (RATS) offers enhanced three-dimensional visualization and superior instrument articulation, enabling precise dissection in confined mediastinal spaces. In this report, we describe a case in which preoperative computed tomography–guided drainage successfully reduced tumor volume, allowing safe and complete resection using RATS.

## Case Report

**Fig. 1:**
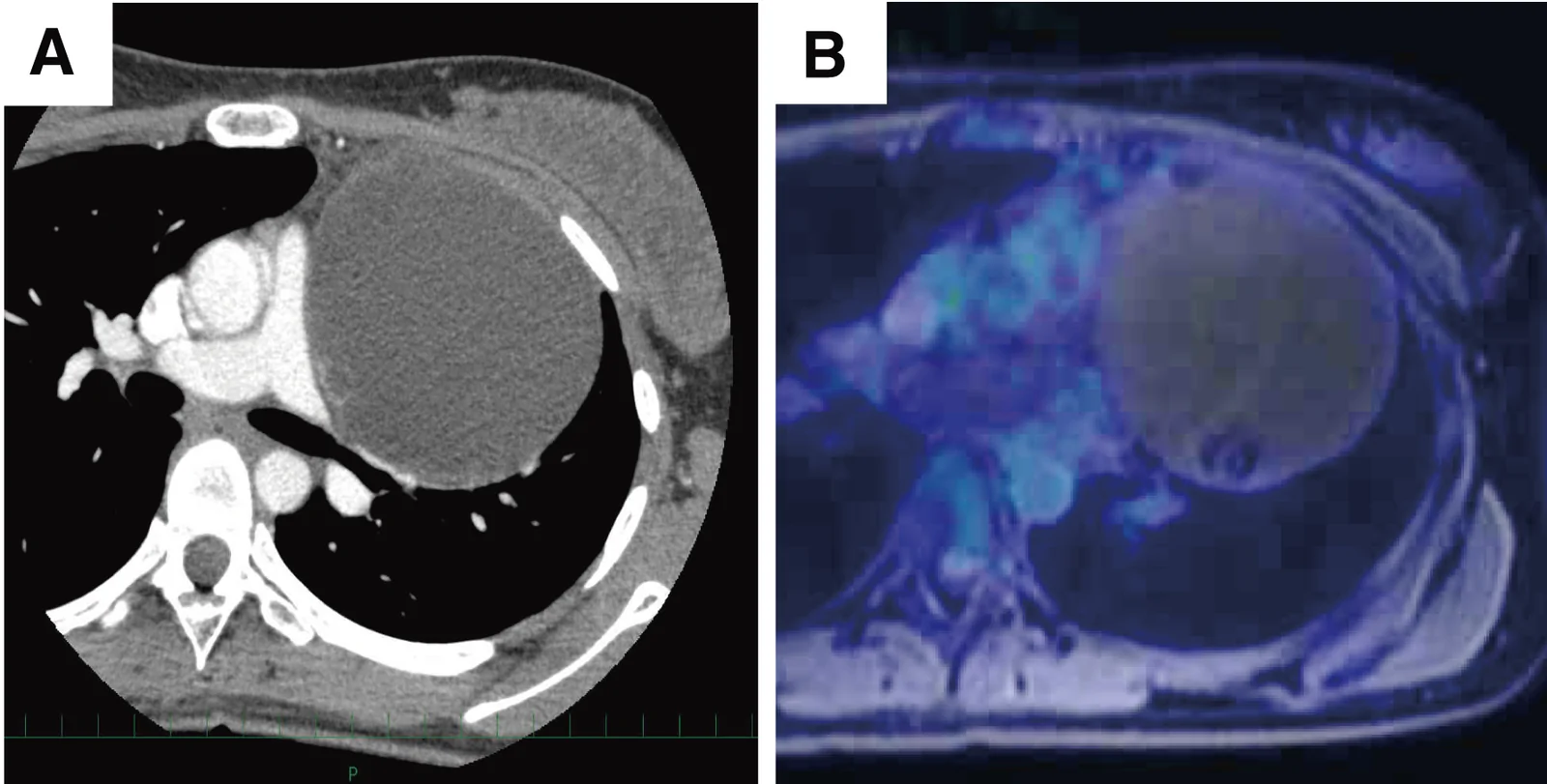
(**A**) Contrast-enhanced chest computed tomography showing an 8.6-cm predominantly cystic anterior mediastinal mass with fat components and minimal enhancement, protruding into the left thoracic cavity and compressing adjacent structures. (**B**) ^18^F-fluorodeoxyglucose positron emission tomography showing no significant uptake, consistent with a benign lesion.

**Fig. 2:**
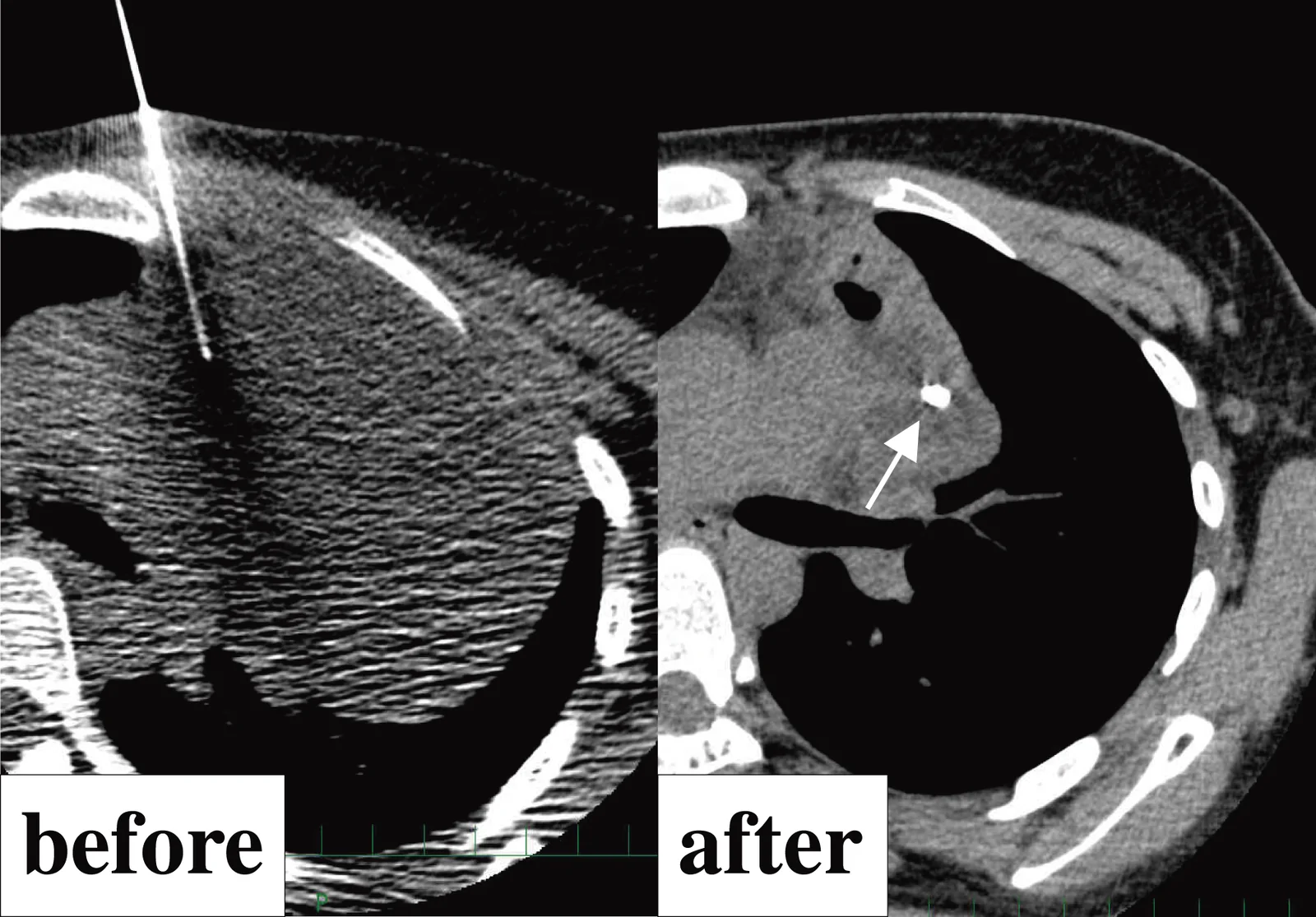
Computed tomography before and after CT-guided transmediastinal drainage. A marked reduction in tumor size is observed after drainage, with expansion of the intrathoracic space. The arrow indicates the indwelling drainage catheter.

A 26-year-old asymptomatic woman was referred to our hospital after an abnormal shadow was detected on routine chest radiography. She had no significant medical history, and serum tumor markers were within normal limits. Contrast-enhanced computed tomography revealed an 8.6-cm predominantly cystic anterior mediastinal mass containing fat components without evidence of invasion. ^18^F-fluorodeoxyglucose positron emission tomography showed no significant uptake, suggesting a benign lesion such as a mature cystic teratoma (**Figs. 1A** and **1B**). Because the imaging findings and normal serum tumor markers strongly suggested a benign mature cystic teratoma and the large tumor size was expected to limit operative exposure and increase the risk of intraoperative spillage, a planned preoperative drainage strategy was adopted. Computed tomography–guided transmediastinal drainage was performed 1 day before surgery using a 7-Fr catheter, and approximately 320 mL of cloudy yellowish fluid was aspirated. Post-drainage imaging demonstrated a reduction in tumor size to approximately 5 cm with improved surgical exposure (**Fig. 2**). RATS was performed the following day via a left-sided approach. The camera port and the second, fourth, and assistant ports were placed in the fifth, sixth, third, and seventh intercostal spaces, respectively, along the anterior axillary line, inframammary line, anterior axillary line, and anterior axillary line. Upon entering the thoracic cavity, dense inflammatory adhesions between the tumor and surrounding structures were encountered and carefully dissected using the robotic system. Immediately before tumor retrieval, the drainage catheter was removed, and the catheter insertion site was immediately sealed by grasping it with a robotic instrument to prevent leakage of cyst contents. The tumor was then placed in a retrieval bag and removed intact without rupture or spillage (**Supplementary Video**). The operative time was 134 minutes with minimal blood loss. The postoperative course was uneventful, and the patient was discharged on postoperative day 4. Histopathological examination confirmed a mature cystic teratoma without malignant components. The patient has remained well during the 12-month follow-up period.

## Discussion

Giant cystic tumors of the anterior mediastinum present a significant challenge for minimally invasive surgery because tumor size limits operative exposure and maneuverability, increasing the risk of intraoperative rupture. In selected cases with a strong suspicion of benign pathology, intraoperative aspiration of cystic contents may improve surgical exposure and facilitate thoracoscopic resection.^1)^ However, intraoperative cyst aspiration carries a risk of intrathoracic spillage, which may lead to contamination and chemical pleuritis, as reported in cases of ruptured mediastinal teratomas.^2)^ In contrast, the preoperative transmediastinal drainage used in the present case enabled tumor decompression prior to intrathoracic manipulation, thereby facilitating safe resection under adequate operative exposure. However, this approach carries a theoretical risk of mediastinal contamination and mediastinitis. This risk may be mitigated by minimizing the interval between drainage and surgery and by performing en bloc resection. Furthermore, in situations requiring meticulous and extensive dissection, RATS may offer advantages over conventional thoracoscopic surgery, including enhanced three-dimensional visualization and improved instrument maneuverability, which may facilitate precise dissection in confined mediastinal spaces.^3)^

Taken together, a staged strategy combining preoperative tumor decompression and robotic surgery may represent a useful approach for achieving safe minimally invasive resection of giant cystic mediastinal tumors. However, careful patient selection and comprehensive preoperative evaluation are essential, as mature cystic teratomas are typically benign but may rarely contain malignant components.^4,5)^ In the present case, imaging findings and tumor markers strongly supported a benign diagnosis, justifying the staged approach with preoperative drainage.

## Conclusion

This case suggests that a staged strategy combining planned preoperative cyst drainage with RATS may facilitate safe and minimally invasive resection of giant benign cystic anterior mediastinal tumors in carefully selected patients.

## Supplementary Materials

Supplementary Video of the surgical procedure. Despite dense adhesions, the tumor was dissected and completely removed in a retrieval bag without rupture or spillage.

## Declarations

### Use of artificial intelligence (AI) tools

The authors used an AI-based tool for English language editing only. The authors take full responsibility for the content.

### Data availability

No new data were created or analyzed in this study. Data sharing is not applicable to this article.
